# Hypofractionated concurrent chemoradiotherapy related lymphopenia and its association with survival in locally advanced non-small cell lung cancer patients

**DOI:** 10.3389/fonc.2022.979384

**Published:** 2022-11-18

**Authors:** FangJie Liu, YingJia Wu, JianHui Shao, Bo Qiu, SuPing Guo, QiaoTing Luo, JinYu Guo, DaQuan Wang, Chu Chu, Rui Zhou, NaiBin Chen, XinLei Ai, Hui Liu

**Affiliations:** ^1^ Department of Radiation Oncology, State Key Laboratory of Oncology in South China, Collaborative Innovation Center for Cancer Medicine, Sun Yat−sen University Cancer Center, Guangzhou, China; ^2^ Guangdong Association Study of Thoracic Oncology, Guangzhou, China

**Keywords:** total lymphocyte count, concurrent chemoradiotherapy, overall survival, locally advanced non-small cell lung cancer, lymphopenia

## Abstract

**Background:**

To evaluate longitudinal changes of concurrent chemoradiotherapy (CCRT) related lymphopenia and its association with survival in locally advanced non-small cell lung cancer (LA-NSCLC) patients.

**Methods:**

Total lymphocyte count (TLC) at baseline, weekly intervals during CCRT and monthly intervals up to 12 months after CCRT were documented. The Common Terminology Criteria for Adverse Events version 5.0 was used to grade the severity of lymphopenia. Cox regression analysis was performed to evaluate the association between overall survival (OS) and CCRT related lymphopenia at different timepoints. Logistic regression model was used to determine the clinical factors associated with TLC level.

**Results:**

381 LA-NSCLC patients treated with definitive CCRT without consolidation therapy (NCT02573506/NCT02577341) between 2011 to 2020 were analyzed. With a median follow-up of 45.8 months, the median OS was 41.0 months for all patients. Univariable analysis demonstrated that the 3 weeks during CCRT Grade (G) 4 lymphopenia (P=0.018), 2 months after CCRT G1-4 lymphopenia (P=0.004), 6 months after CCRT (6m-post-CCRT) G1-4 lymphopenia (P=0.001), and TLC nadir (P=0.020) were significantly associated with poorer OS. Multivariable analysis suggested that 6m-post-CCRT G1-4 lymphopenia (HR 2.614; P=0.041) were one of the independent predictors of OS. Further analysis inferred that radiation dose (OR: 1.328; P=0.005), GTV volume (OR: 1.004; P=0.036), and baseline TLC (OR: 0.288; P=0.001) were associated with 6m-post-CCRT lymphopenia.

**Conclusion:**

The persistent lymphopenia at 6 months after CCRT was an independent prognostic factor of OS in LA-NSCLC patients. Higher radiation dose, larger gross tumor volume and lower baseline TLC were significantly related to 6m-post-CCRT lymphopenia.

## Introduction

Lymphocytes play a crucial role in the host antitumor immune response, which helps suppress cancer progression. Total lymphocyte count (TLC) has been recognized as an important prognostic factor for multiple solid tumors in clinical studies (1, 2). Although concurrent chemoradiotherapy (CCRT) is a standard treatment modality for unresectable locally advanced non-small cell lung cancer (LA-NSCLC), it frequently results in lymphopenia from directly destructing mature circulating lymphocytes, or interfering lymphopoiesis in radiation-sensitive organs, such as bone marrow (3, 4). The reduction of TLC caused by CCRT is associated with poor prognosis in various cancers (5–8). Previous studies have demonstrated that lymphopenia was a predictor of inferior survival as well as acute/chronic inflammatory pneumonitis among patients with NSCLC (7, 9, 10). Nevertheless, the dynamic change of TLC during and after CCRT and its role on prognosis has not been fully elucidated in NSCLC.

CCRT with consolidative immunotherapy offers the best chance for cure in patients with unresectable LA-NSCLC. Moreover, ex vivo studies suggested that radiotherapy can exert immunostimulatory effects through increased expression of cytokines and tumor associated antigens, and through recruitment of effector cells into tumor microenvironment (4, 11). However, CCRT induced lymphopenia might have negative impact on NSCLC patients receiving immunotherapy. Previous study also indicated that lymphopenia could be regarded as an early indicator of immune-related adverse events (12), and severe lymphopenia at the time of immunotherapy initiation was an independent predictor of worse progression-free survival (PFS) (13). Therefore, to explore the longitudinal changes of TLC during and after CCRT is crucial for maximizing the efficacy of combined therapy. Clinical factors associated with persistent lymphopenia and strategies to mitigate lymphopenia effects should be explored.

Given the association between TLC and survival in multiple solid tumors as well as the important role of the immune response in patients with LA-NSCLC, we hypothesized that not only the TLC nadir during CCRT but also the persistent lymphopenia after CCRT could negatively affect survival outcomes in patients with LA-NSCLC treated with CCRT. The objectives of this study were two-fold: (a) to investigate the relationship between longitudinal TLC changes and survival outcome in LA-NSCLC patients undergoing definitive CCRT and (b) to evaluate the clinical factors associated with persistent lymphopenia induced by CCRT in the patient population.

## Materials and methods

### Patient population

A total of 381 patients diagnosed as unresectable LA-NSCLC and treated with definitive CCRT were identified, including the patient cohorts from two clinical trials (NCT02573506, NCT02577341) as well as patients treated as the protocol of the trials. The inclusive criteria were (1): biopsy-proven NSCLC; (2) stage IIIA-IIIC according to the eighth edition of the International Union against Cancer/American Joint Committee on Cancer staging system. (3) receipt of CCRT with curative intent; (4) without adjuvant or consolidation therapy after CCRT; (5) age of 18 years or older; (6) Eastern Cooperative Oncology Group (ECOG) performance status score of 0 to 2. (7) complete blood count (CBC) with differential available prior to CCRT, weekly intervals during CCRT and monthly intervals up to 12 months after CCRT. Patients were excluded if they had metastatic disease at the time of diagnosis. Patient and disease characteristics including age, gender, ECOG score, family history of cancer, smoking history, histologic grade, TNM staging, pre-CCRT body mass index (BMI), pre-CCRT albumin were documented. Radiotherapy regimen information including gross tumor volume (GTV), total radiation dose, mean body dose (MBD), mean heart dose (MHD), and mean lung dose (MLD) were collected.

This study was approved by the review board of Sun Yat-sen University Cancer Center. Written informed consent for the use of clinical data was obtained from all patients.

### Treatment

All the patients underwent definitive CCRT. A four-dimensional simulation CT was created and the GTV were composite volumes from CT scans of all breathing phases. The GTV was defined as the visible primary tumor and positive lymph nodes on CT scans. The planning target volumes for GTV were created by a uniform expansion of 5-6 mm surrounding the GTV for all patients. Involved nodal irradiation was adopted in our patients. Definitive CCRT was prescribed per clinical trial protocols (NCT02573506, NCT02577341). Radiotherapy was delivered using intensity modulated radiotherapy technique with a total dose of 60-68 Gy to PTV-GTV in 16-23 daily fractions. Concurrent chemotherapy regimens included docetaxel/paclitaxel plus platinum (14, 15). Three to five cycles of weekly concurrent chemotherapy were delivered depending on the fractions.

### Longitudinal complete blood count

White blood cell, neutrophil, hemoglobin, and TLC prior to CCRT, weekly intervals during CCRT and monthly intervals up to 12 months after CCRT were documented. Longitudinal TLCs, including the baseline (pre-CCRT) TLC (within 2 weeks prior to RT), the 3 weeks during CCRT (mid-CCRT) TLC, the end of CCRT (end-CCRT) TLC, 2 months after CCRT (2m-post-CCRT) TLC, 6 months after CCRT (6m-post-CCRT) TLC and TLC nadir, were collected for analysis. TLC nadir was recorded as the minimum value from the onset till 12 months after CCRT. As treatment beyond progression was supposed to affect TLC, 2m-post-CCRT TLC and 6m-post-CCRT TLC were recorded for those patients who were progression-free for at least 6 months after CCRT. The Common Terminology Criteria for Adverse Events (CTCAE) version 5.0 was used to grade the severity of lymphopenia (G0: ≥1.00 k/µL, G1: <1.00-0.80 k/µL, G2: <0.80–0.50 k/µL, G3: <0.50–0.20 k/µL, and G4: <0.20 k/µL).

### Follow-up

Patients were firstly followed up every 3 months during the first 2 years, every 6 months in 3-5 years, and every year thereafter until progression or death. Medical history, physical examination, CT scans of the chest and upper abdomen were performed at each follow-up. Brain magnetic resonance imaging, bone scan or positron emission tomography/CT were performed when tumor recurrence or metastasis was suspected.

### Statistical analysis

The characteristics of patients, disease and treatments were summarized by descriptive analysis. Continuous variables were presented with median (range) and compared by Mann-Whitney U test. The primary endpoint of the study was overall survival (OS) which calculated from the date of initial treatment to death of any cause or time of last follow-up. Progression free survival was calculated as the time interval between the initial treatment to the date of disease progression or death. OS, PFS and local control were analyzed using the Kaplan-Meier method and log-rank test. Univariable and multivariable Cox regression model were performed to select the independent predictors of OS. The hazard ratio (HR) with their 95% confidence interval (CI) were calculated. Clinical and dosimetric factors were analyzed by univariable and multivariable binary logistic regression model to identify independent baseline variables associated with 6m-post-CCRT lymphopenia. The odds ratio (OR) with their 95% CI were calculated. All data were analyzed using the SPSS statistics, version 26.0 (IBM Corp., Armonk, NY). Two-tailed *P* value <0.05 was considered statistically significant.

## Results

### Patient characteristics

The baseline characteristics of 381 patients are summarized in **Table 1**. For the entire patient cohort, the median age was 58 (range, 28-81). The majority were male (82.2%) and had an ECOG performance status of 0-1 (92.4%). At diagnosis, 59.8% of patients had a smoking history. There were 208 (54.6%) patients had squamous cell carcinoma, and 194 (50.9%) patients had stage IIIB disease. The median radiation dose was 65.78 Gy (range, 59.80-68.00). Patients received weekly concurrent chemotherapy (25 mg/m^2^ docetaxel and 25 mg/m^2^ platinum) at 3~5 cycles. The median GTV volume was 98.5 cm^3^ (range, 21.5-664.3). The median values of MBD, MHD and MLD were 8.59 Gy (range, 2.27-16.57), 11.89 Gy (range, 0.74-45.20), and 18.87 Gy (range, 2.81-27.96), respectively.

**Table 1:** Characteristics of patients (n=381).

	Total
Characteristics	(N=381)
Age, median (yr, range)	58 (28-81)
Gender, n (%)	
Male	313 (82.2)
Female	68 (17.8)
ECOG, n (%)	
0	88 (23.1)
1	264 (69.3)
2	29 (7.6)
Smoking history, n (%)	
Yes	228 (59.8)
No	153 (40.2)
Family history of cancer, n (%)	
Yes	81 (21.3)
No	300 (78.7)
Histology, n (%)	
Adenocarcinoma	130 (34.1)
Squamous cell carcinoma	208 (54.6)
Others	43 (11.3)
Disease stage, n (%)	
IIIA	108 (28.3)
IIIB	194 (50.9)
IIIC	79 (20.8)
Total dose, median (Gy, range)	65.78 (59.80-68.00)
GTV volume, median (cm^3^, range)	98.5 (21.5-664.3)
Concurrent chemotherapy (cycles)	
3	137 (36.0)
4	214 (56.2)
5	30 (7.9)
Pre-CCRT TLC, median (k/μL, range)	1.70 (0.40-3.60)
Pre-CCRT BMI, n (%)	
<18.5 kg/m^2^	21 (5.8)
≥18.5 kg/m^2^	338 (94.2)
Pre-CCRT albumin, n (%)	
<35 g/L	11 (3.0)
≥35 g/L	357 (97.0)
Pre-CCRT neutrophil, n (%)	
<1.8 k/μL	23 (6.1)
≥1.8 k/μL	353 (93.9)
Pre-CCRT hemoglobin, n (%)	
<100 g/L	16 (4.3)
≥100 g/L	360 (95.7)
MBD, median (Gy, range)	8.59 (2.27-16.57)
MHD, median (Gy, range)	11.89 (0.74-45.20)
MLD, median (Gy, range)	18.87 (2.81-27.96)

BMI, body mass index; ECOG, Eastern Cooperative Oncology Group; GTV, gross tumor volume; MBD, mean body dose; MHD, mean heart dose; MLD, mean lung dose; Pre-CCRT, before concurrent chemoradiotherapy; TLC, total lymphocyte count.

### Longitudinal total lymphocyte counts

**Figure 1:**
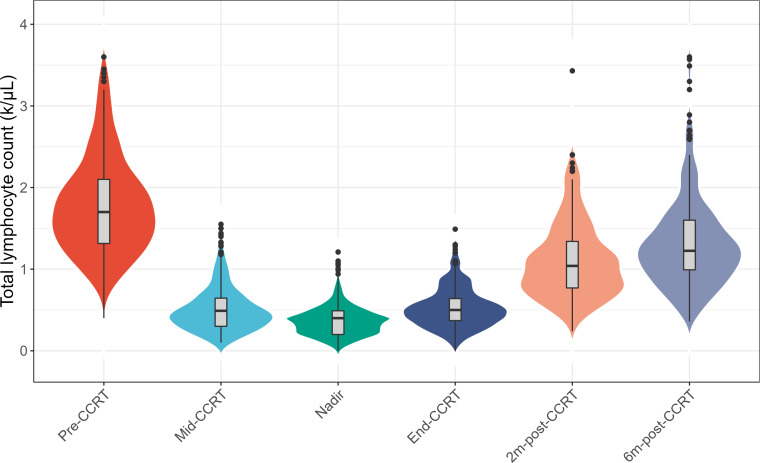
Changes of the total lymphocyte count (TLC) at longitudinal timepoints during concurrent chemoradiotherapy (CCRT) and follow-up. Abbreviations: pre-CCRT, the baseline; mid-CCRT, the 3 weeks during CCRT; end-CCRT, the end of CCRT; 2m-post-CCRT, 2 months after CCRT; 6m-post-CCRT, 6 months after CCRT.

The longitudinal dynamic change of TLC was shown in **Figure 1**. The median pre-CCRT TLC was 1.70 k/μL (range, 0.40-3.60). At 3 weeks during CCRT (mid-CCRT), the median TLC was 0.49 k/μL (range, 0.10-1.55), and the rate of G4 lymphopenia was 5.0%. The median end-CCRT TLC was 0.50 k/μL (range, 0-1.49), and the rate of G4 lymphopenia was 3.2%. The median 2m-post-CCRT TLC was 1.04 k/μL (range, 0.24-3.43), however, 46.4% of patients had G1-4 lymphopenia. The median 6m-post-CCRT TLC was 1.22 k/μL (range, 0.36-3.60). 75.0% of patients had TLC returned to >1.0 k/μL (G0) by 6 months, whereas 25.0% had persistent lymphopenia beyond 6 months. The median TLC nadir was 0.40 k/μL (range, 0-1.21), and 10.8% (41/381) of patients experienced G4 lymphopenia during the whole course of CCRT (**Table 2**).

**Table 2:** The association between TLC and overall survival (OS).

Factor	No. of patients (%)	Stratification	No. of patients (%)	Median OS (95% CI)	*P^a)^*
Pre-CCRT TLC					
G0	359 (94.5)				
G1	17 (4.5)	G0	359 (94.5)	42.5 (35.2-49.9)	0.371
G2	3 (0.8)	G1-4	21 (5.5)	34.4 (27.3-41.5)	
G3	1 (0.2)				
G4	0 (0)				
Mid-CCRT TLC					
G0	32 (8.5)				
G1	28 (7.5)	G0-3	356 (95.0)	41.9 (33.8-50.0)	**0.016**
G2	124 (33.1)	G4	19 (5.0)	27.7 (11.0-44.3)	
G3	172 (45.9)				
G4	19 (5.0)				
End-CCRT TLC					
G0	18 (5.9)				
G1	33 (10.7)	G0-3	297 (96.8)	44.9 (36.1-53.6)	0.108
G2	119 (38.8)	G4	10 (3.2)	16.8 (0-39.0)	
G3	127 (41.4)				
G4	10 (3.2)				
2m-post-CCRT TLC					
G0	140 (53.6)				
G1	49 (18.8)	G0	140 (53.6)	52.8 (36.7-69.0)	**0.004**
G2	64 (24.5)	G1-4	121 (46.4)	38.3 (23.8-52.8)	
G3	8 (3.1)				
G4	0 (0)				
6m-post-CCRT TLC					
G0	183 (75.0)				
G1	30 (12.3)	G0	183 (75.0)	54.0 (41.4-66.6)	**0.001**
G2	28 (11.5)	G1-4	61 (25.0)	28.8 (24.6-33.0)	
G3	3 (1.2)				
G4	0 (0)				
TLC nadir					
G0	5 (1.3)				
G1	10 (2.6)	G0-3	340 (89.2)	41.9 (33.9-49.8)	**0.019**
G2	79 (20.7)	G4	41 (10.8)	34.4 (10.1-58.7)	
G3	246 (64.6)				
G4	41 (10.8)				

a) Analyzed by log-rank test between two subgroups.

pre-CCRT TLC, TLC before concurrent chemoradiotherapy (CCRT); mid-CCRT TLC, TLC at the 3 weeks in CCRT; end-CCRT TLC, TLC at the end of CCRT; 2m-post-CCRT TLC, TLC at 2 months after CCRT; 6m-post-CCRT TLC, TLC at 6 months after CCRT.

The bold values mean the data were considered statistically significant as the P value <0.05.

### Clinical and dosimetric factors associated with overall survival

**Figure 2:**
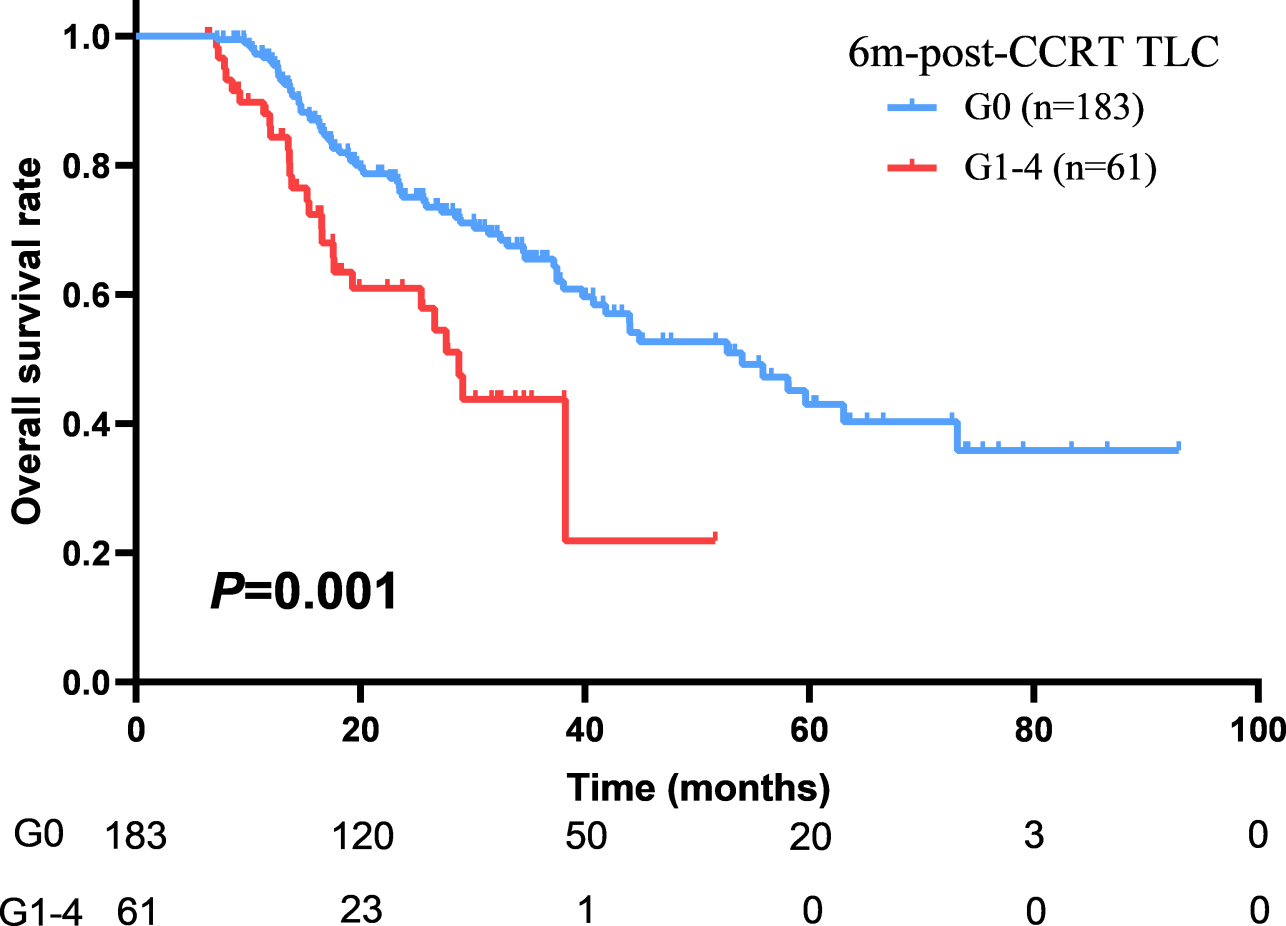
The association between overall survival (OS) and 6m-post-CCRT TLC.

**Figure 3:**
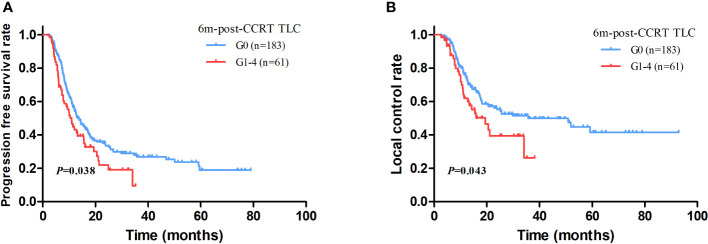
The association between progression free survival (PFS) **(A)** and local control **(B)** with 6m-post-CCRT TLC.

With a median follow up of 45.8 months (range: 1.5-92.9 months), the median OS and PFS were 41.0 months (95%CI: 36.2–45.9 months) and 12.7 months (95%CI: 11.3–14.2 months) for all patients. The 1-year and 2-year of local control rate were 70.2% and 50.9%, respectively. Age (HR: 1.542; *P*=0.005), gender (HR: 1.548; *P*=0.046), ECOG (HR: 1.496; *P*=0.008), disease stage (HR: 1.325; *P*=0.010), radiation dose (HR: 1.083; *P*=0.037), GTV volume (HR: 1.004; *P*<0.001), mid-CCRT TLC (HR: 2.040; *P*=0.018), 2m-post-CCRT TLC (HR: 1.754; *P*=0.004), 6m-post-CCRT TLC (HR: 2.189; *P*=0.001), TLC nadir (HR: 1.655; *P*=0.020), MBD (HR: 1.110; *P*=0.002), and MLD (HR: 1.085; *P*=0.001) were significantly associated with OS on univariable Cox regression analysis. Subsequently multivariable analysis demonstrated that gender (HR: 3.797; *P*=0.009), disease stage (HR: 1.620; *P*=0.045), GTV volume (HR: 1.004; *P*=0.035), 6m-post-CCRT TLC (HR: 2.614; *P*=0.041) were independent predictors of OS (**Table 3**, **Figure 2**). The relationship between progression free survival and local control rates with 6m-post-CCRT TLC were shown in **Figure 3**.

**Table 3:** Univariable and multivariable Cox regression analysis of potential variables associated with OS in LANSCLC patients.

	Univariable analysis		Multivariable analysis	
Variables	HR (95% CI)	*P*	HR (95% CI)	*P*
Age (≥58)	1.542 (1.142-2.081)	**0.005**	1.707 (0.888-3.282)	0.109
Gender (Male)	1.548 (1.007-2.381)	**0.046**	3.797 (1.386-10.400)	**0.009**
ECOG	1.496 (1.112-2.011)	**0.008**	1.456 (0.824-2.571)	0.196
Smoking history (Smoker)	1.360 (0.999-1.851)	0.051		
Family history of cancer (Yes)	0.870 (0.601-1.259)	0.460		
Histology	0.921 (0.733-1.157)	0.921		
Disease stage	1.325 (1.071-1.640)	**0.010**	1.620 (1.010-2.599)	**0.045**
Radiation dose (Continuous)	1.083 (1.005-1.166)	**0.037**	1.003 (0.871-1.156)	0.966
GTV volume (Continuous)	1.004 (1.003-1.005)	**<0.001**	1.004 (1.000-1.007)	**0.035**
Pre-CCRT TLC (G1-4)	1.321 (0.716-2.436)	0.373		
Mid-CCRT TLC (G4)	2.040 (1.131-3.680)	**0.018**	0.867 (0.123-6.090)	0.886
End-CCRT TLC (G4)	1.937 (0.852-4.405)	0.115		
2m-post-CCRT TLC (G1-4)	1.754 (1.194-2.576)	**0.004**	1.233 (0.564-2.699)	0.600
6m-post-CCRT TLC (G1-4)	2.189 (1.366-3.508)	**0.001**	2.614 (1.040-6.573)	**0.041**
TLC nadir (G4)	1.655 (1.082-2.532)	**0.020**	1.879 (0.511-6.907)	0.343
Pre-CCRT BMI (<18.5)	1.559 (0.901-2.697)	0.112		
Pre-CCRT albumin (<35)	0.807 (0.356-1.833)	0.609		
Pre-CCRT neutrophil (<1.8)	1.047 (0.568-1.932)	0.883		
Pre-CCRT hemoglobin (<100)	1.361 (0.695-2.668)	0.369		
MBD (Continuous)	1.110 (1.039-1.185)	**0.002**	1.130 (0.993-1.286)	0.064
MHD (Continuous)	1.014 (0.993-1.034)	0.186		
MLD (Continuous)	1.085 (1.032-1.140)	**0.001**	1.049 (0.922-1.193)	0.470

BMI, body mass index; CI: confidence interval; ECOG, Eastern Cooperative Oncology Group; GTV, gross tumor volume; HR, hazard ratio; pre-CCRT TLC, TLC before concurrent chemoradiotherapy (CCRT); mid-CCRT TLC, TLC at the 3 weeks in CCRT; end-CCRT TLC, TLC at the end of CCRT; 2m-post-CCRT TLC, TLC at 2 months after CCRT; 6m-post-CCRT TLC, TLC at 6 months after CCRT; pre-CCRT, before concurrent chemoradiotherapy; MBD, mean body dose; MHD, mean heart dose; MLD, mean lung dose.

The bold values mean the data were considered statistically significant as the P value <0.05.

### Clinical and dosimetric factors related to 6m-post-CCRT lymphopenia

Clinical and dosimetric factors affecting 6m-post-CCRT lymphopenia were shown in **Table 4**. Total radiation dose (OR: 1.307; *P*=0.002), GTV volume (OR: 1.005; *P*=0.002), pre-CCRT TLC (OR: 0.339; *P*<0.001), pre-CCRT BMI (OR: 3.044, *P*=0.040), and MLD (OR: 1.118, *P*=0.027) were found to be significantly associated with 6m-post-CCRT lymphopenia on the univariate analysis. Subsequently multivariable analysis revealed that radiation dose (OR: 1.328; *P*=0.005), GTV volume (OR: 1.004; *P*=0.036), and pre-CCRT TLC (OR: 0.288; *P*=0.001) were independent predictors of persistent lymphopenia at 6 months after CCRT.

**Table 4:** Binary logistic regression results of variables related to persistent lymphopenia at 6 months after CCRT.

	n (%)	6m-post-CCRT lymphopenia		OR	OR
Variables	/Median (range)	G0 (n=183)	G1-4 (n=61)	(univariable)	(multivariable)
Age (yrs)	Median (range)	56 (28-77)	57 (34-81)	0.997 (0.967-1.028, p=0.856)	
Gender	female	32 (17.5)	14 (23.0)	–	
	Male	151 (82.5)	47 (77.0)	0.711 (0.350-1.445, p=0.346)	
ECOG (2 vs. 1 vs. 0)	0	51 (27.8)	20 (32.8)	–	
	1	118 (64.5)	38 (62.3)	0.821 (0.436-1.547, p=0.542)	
	2	14 (7.7)	3 (4.9)	0.546 (0.142-2.108, p=0.380)	
Smoking history	Non-smoker	75 (41.0)	32 (52.5)	–	
	Smoker	108 (59.0)	29 (47.5)	0.629 (0.351-1.127, p=0.119)	
Family history of cancer	No	142 (77.6)	49 (80.3)	–	
	Yes	41 (22.4)	12 (19.7)	0.848 (0.413-1.744, p=0.654)	
Histology	adenocarcinoma	70 (38.3)	18 (29.5)	–	
	squamous cell carcinoma	92 (50.3)	34 (55.7)	1.437 (0.750-2.754, p=0.274)	
	Others	21 (11.4)	9 (14.8)	1.667 (0.653-4.254, p=0.285)	
Disease stage	IIIA	49 (26.8)	18 (29.5)	–	
	IIIB	100 (54.6)	27 (44.3)	0.735 (0.370-1.462, p=0.380)	
	IIIC	34 (18.6)	16 (26.2)	1.281 (0.574-2.860, p=0.546)	
Radiation dose (Gy)	Median (range)	65.78 (59.80-68.00)	68.00 (62.79-68.00)	1.307 (1.103-1.547, **p=0.002**)	1.328 (1.092-1.614, **p=0.005**)
GTV volume (cm^3^)	Median (range)	94.0 (21.5-503.3)	134.4 (23.1-567.7)	1.005 (1.002-1.008, **p=0.002**)	1.004 (1.000-1.007, **p=0.036**)
Concurrent chemotherapy (cycles)	3	75 (41.0)	21 (34.4)	–	
	4	96 (52.4)	39 (63.9)	1.451 (0.788-2.671, p=0.232)	
	5	12 (6.6)	1 (1.7)	0.298 (0.037-2.422, p=0.257)	
Pre-CCRT TLC (k/μL)	Median (range)	1.80 (0.80-3.60)	1.50 (0.60-3.05)	0.339 (0.185-0.622, **p<0.001**)	0.288 (0.137-0.605, **p=0.001**)
Pre-CCRT BMI (kg/m^2^)	≥18.5	167 (95.4)	48 (87.3)	–	
	<18.5	8 (4.6)	7 (12.7)	3.044 (1.051-8.822, **p=0.040**)	0.133
Pre-CCRT albumin (g/L)	≥35	176 (97.2)	60 (98.4)	–	
	<35	5 (2.8)	1 (1.6)	0.587 (0.067-5.122, p=0.630)	
Pre-CCRT neutrophil (k/μL)	≥1.8	170 (93.4)	59 (96.7)	–	
	<1.8	12 (6.6)	2 (3.3)	0.480 (0.104-2.209, p=0.346)	
Pre-CCRT hemoglobin (g/L)	≥100	174 (95.6)	58 (95.1)	–	
	<100	8 (4.4)	3 (4.9)	1.125 (0.289-4.382, p=0.865)	
MBD (Gy)	Median (range)	8.04 (2.27-16.57)	8.25 (4.47-13.58)	1.071 (0.942-1.217, p=0.296)	
MHD (Gy)	Median (range)	11.51 (1.14-45.20)	11.20 (2.23-35.37)	1.006 (0.965-1.048, p=0.782)	
MLD (Gy)	Median (range)	18.32 (2.81-26.48)	19.45 (10.93-25.46)	1.118 (1.013-1.235, **p=0.027**)	0.833

BMI, body mass index; ECOG, Eastern Cooperative Oncology Group; GTV, gross tumor volume; pre-CCRT, before concurrent chemoradiotherapy; MBD, mean body dose; MHD, mean heart dose; MLD, mean lung dose; OR, odds ratio; TLC, total lymphocyte count.

The bold values mean the data were considered statistically significant as the P value <0.05.

## Discussion

The study investigated the relationship between the longitudinal TLC changes and survival outcome in LA-NSCLC patients undergoing definitive CCRT and evaluated the clinical factors associated with persistent lymphopenia induced by CCRT in the patient population. The results of current study suggested that the 6m-post-CCRT persistent lymphopenia was the independent predictor of OS among different time points.

94.5% of LA-NSCLC patients had a G0 TLC before CCRT, which dropped to 53.6% and 75.0% at 2 months and 6 months after the completion of CCRT. At 6 months after CCRT, 25% of patients had G1-4 CCRT related lymphopenia, with 1.2% of patients had G3 lymphopenia and no patients had G4 lymphopenia. The degree and timing of CCRT related lymphopenia in our study were consistent with previous studies that 51%-84% of patients had TLC reduction, the incidence of G3-4 lymphopenia was 35%-61% at 2 months after CCRT and persisted throughout one year of observation (5, 16–19).

As lymphocyte plays a vital role in infiltrating and destroying tumor tissues by activation of cellular immune response through T cells, TLC may be recognized as one of biomarkers to represent the immune status and anti-tumor response. Radiation to those circulating blood pool such as heart and lung might reduce tumor infiltrating lymphocytes by depleting circulating lymphocyte subpopulations, including CD8+ cytotoxic T cells, CD4+ helper T cells, and Foxp3+ regulatory T cells which have been proved to strongly correlate with survival outcome in NSCLC. Previous studies found that the TLC nadir, as well as lymphopenia occurred in 1 and 3 months after radiotherapy were significantly associated with OS in limited stage small cell lung cancer (20, 21). A large retrospective analysis of 901 patients with NSCLC demonstrated that TLC nadir with G3-4 lymphopenia was an independent factor of OS (11). Our results also found that TLC nadir was significantly associated with inferior OS in univariable analysis. Moreover, the multivariable analysis of current study suggested that the 6m-post-CCRT lymphopenia was the independent predictor of OS, indicating that the persistent lymphopenia after CCRT was crucial for long-term treatment outcome and regular examination was important for LA-NSCLC patients. The exact mechanism by which CCRT related lymphopenia affects OS is not clear. The impaired anti-tumor immunity associated with lymphopenia is one possible explanation. Recent studies suggested higher tumor infiltrated lymphocytes were associated with better disease control in lung cancer patients treated with immunotherapy (22, 23). In addition, CCRT related lymphopenia may lead to poorer OS due to increased lymphopenia-related complications such as infection (18). In our study, 103 patients (27.0%) used antibiotics for infection or ≥G2 radiation pneumonitis. In those patients, 3 patients (0.8%) experienced a sepsis, and 46 patients (12.1%) experienced infection confirmed by the procalcitonin testing and sputum culture and treated with prolonged antibiotics. For patients who had recurrent infections were more likely to have prolonged antibiotic use, which may induce antibiotic-associated bone marrow suppression through depletion of gut microbiota (24). In this case, lymphopenia as the reflector of myelosuppression was thought to be another prognostic factor for those patients.

PACIFIC study established the role of consolidative immunotherapy after CCRT for unresectable LA-NSCLC. However, not all patients could benefit from this strategy, raising an urgent need to discover important predictive factors. Among them, radiation induced lymphopenia is worthy of attention. Lymphopenia has been shown to negatively impact the treatment outcome of immunotherapy. Yeona et al. (13) demonstrated that patients with peri-immunotherapy lymphopenia showed worse OS and PFS. Friedes et al. (25) found that G3-4 lymphopenia before immunotherapy was associated with disease progression in patients with LA-NSCLC receiving consolidative immunotherapy after CCRT. Therefore, monitoring the recovery of TLC after CCRT was clinically important. Patients with favorable TLC recovery after CCRT and persistent normal TLC during long-term follow up could benefit more from the Pacific paradigm. In the current study, 46.4% and 25.0% of patients had persistent G1-4 lymphopenia at 2 months and 6 months after CCRT, respectively. Considering the impact of persistent lymphopenia on treatment outcome, utilizing TLC as a stratification tool for the initiation time of immunotherapy might help to improve treatment efficacy.

As the 6m-post-CCRT lymphopenia was independently associated with OS, pretreatment clinical factors associated with lymphopenia after CCRT and strategies to reduce the incidence of persistent lymphopenia should be considered. A growing number of studies have demonstrated that the incidence and severity of lymphopenia is associated with GTV volume, duration of radiotherapy, radiation fraction, multiple irradiated sites and receipt of concurrent chemotherapy (13, 26, 27). Abravan et al. has reported that vertebrae V20, MLD and MHD as predictors of lymphopenia by data mining approach (11). Higher doses of radiation to the immune system were associated with the development of G3 lymphopenia, tumor progression and death after the definitive treatment of stage III NSCLC (28). In our study, radiation dose, GTV volume, and pre-CCRT TLC were identified as significantly predictors of persistent lymphopenia. These results indicated that the appropriate radiation dose to gross tumor, effective induction therapy to reduce tumor volume before CCRT and nutrition support to maintain TLC level might be essential for clinical practice.

This study has several limitations aside from its retrospective nature. Firstly, some dosimetric factors were not included in the analysis, such as lung V5, and heart V5 (10, 29). There might be other dosimetric factors which contribute to the recovery of TLCs. Secondly, we included patients irradiated at different doses and fractionations, that might have different impact on the results. Third, all the patients in current study had no consolidative immunotherapy. For patients treated with CCRT followed by consolidation immunotherapy, the TLC after CCRT was supposed to be different and warrants further investigation.

In conclusion, the persistent G1-4 lymphopenia at 6 months after CCRT was found to be a prognostic factor of OS in LA-NSCLC patients. Higher radiation dose, larger gross tumor volume and lower baseline TLC were significantly related to 6m-post-CCRT lymphopenia. The association between the CCRT relate lymphopenia and OS suggests an important role of the host immunity in LA-NSCLC outcomes.

## Data availability statement

The raw data supporting the conclusions of this article will be made available by the authors, without undue reservation.

## Ethics statement

The studies involving human participants were reviewed and approved by the review board of Sun Yat-sen University Cancer Center. The patients/participants provided their written informed consent to participate in this study.

## Author contributions

Study conception and design: FL, YW, JS, and HL. Literature review: BQ. Data acquisition: YW, QL, FL, SG, JS, JG, DW, CC, and RZ. Statistical analysis: NC, XA, HL, and BQ. Data interpretation: FL, YW and JS. Manuscript preparation: FL, YW, JS, and HL. Manuscript review: All authors. All authors contributed to the article and approved the submitted version.

## Funding

This study was supported by the National Natural Science Foundation of China (Grant Number 82073328).

## Conflict of interest

The authors declare that the research was conducted in the absence of any commercial or financial relationships that could be construed as a potential conflict of interest.

## Publisher’s note

All claims expressed in this article are solely those of the authors and do not necessarily represent those of their affiliated organizations, or those of the publisher, the editors and the reviewers. Any product that may be evaluated in this article, or claim that may be made by its manufacturer, is not guaranteed or endorsed by the publisher.
